# CRISPR-based transcriptional activation deciphers strigolactone biosynthesis in *Nicotiana benthamiana*

**DOI:** 10.1093/plphys/kiag748

**Published:** 2026-10-06

**Authors:** Juan Pablo Arciniegas, Derry Alvarez, Jian You Wang, Marta Vazquez-Vilar, Diego Orzaez, Salim Al-Babili

**Affiliations:** The BioActives Lab, King Abdullah University of Science and Technology, P.O. Box 4700, Thuwal 23955-6900, Saudi Arabia; Plant Science Program, Biological and Environmental Science and Engineering (BESE) Division, King Abdullah University of Science and Technology (KAUST), P.O. Box 4700, Thuwal 23955-6900, Saudi Arabia; The BioActives Lab, King Abdullah University of Science and Technology, P.O. Box 4700, Thuwal 23955-6900, Saudi Arabia; Plant Science Program, Biological and Environmental Science and Engineering (BESE) Division, King Abdullah University of Science and Technology (KAUST), P.O. Box 4700, Thuwal 23955-6900, Saudi Arabia; The BioActives Lab, King Abdullah University of Science and Technology, P.O. Box 4700, Thuwal 23955-6900, Saudi Arabia; Academia Sinica Biotechnology Center In Southern Taiwan, Agricultural Biotechnology Research Center, Academia Sinica, Tainan 71110, Taiwan; Institute of Tropical Plant Sciences and Biology, National Cheng Kung University, Tainan 701401, Taiwan; Instituto de Biología Molecular y Celular de Plantas (IBMCP), Consejo Superior de Investigaciones Científicas, Universidad Politécnica de Valencia, 46022 Valencia, Spain; Instituto de Biología Molecular y Celular de Plantas (IBMCP), Consejo Superior de Investigaciones Científicas, Universidad Politécnica de Valencia, 46022 Valencia, Spain; The BioActives Lab, King Abdullah University of Science and Technology, P.O. Box 4700, Thuwal 23955-6900, Saudi Arabia; Plant Science Program, Biological and Environmental Science and Engineering (BESE) Division, King Abdullah University of Science and Technology (KAUST), P.O. Box 4700, Thuwal 23955-6900, Saudi Arabia

Dear Editor,

Strigolactones (SLs) are carotenoid-derived phytohormones involved in different developmental and adaptive processes, as well as in the rhizospheric communication with root parasitic plants and mycorrhizal fungi. To date, there are more than 40 natural identified SLs with the characteristic butenolide ring, which can be divided into canonical and noncanonical SLs, based on the presence and absence of a tricyclic lactone moiety (ABC-ring), respectively (Wang et al. 2024). This structural diversity reflects a functional diversification and results from a complex biosynthesis pathway that involves different sorts of enzymes, including isomerases, carotenoid cleavage dioxygenases (CCDs), cytochrome P450s (CYPs), methyltransferases, and 2-oxoglutarate Fe (II) dependent dioxygenase (2OGs), besides yet unknown enzymes (Jia et al. 2018; Wang, et al. 2024). To functionally characterize the enzymes, various platforms have been implemented, including *in vitro* enzymatic assays involving protein production in *E. coli* or CYPs expression in yeast microsomes (Zhou et al. 2025b), as well as reconstitution of the SL biosynthetic pathway in bacterium-yeast consortia (Zhou et al. 2025a). Additionally, transient expression in *Nicotiana benthamiana* has been widely used to determine the function of SL biosynthesis-related enzymes from various plant species. However, knowledge about the SL pathway in *N. benthamiana* remains limited.

A previous report confirmed the core pathway in *N. benthamiana*, comprising DWARF27 (D27), CCD7, and CCD8, which leads to carlactone (CL), the central intermediate and precursor of both canonical and noncanonical SLs (Yata et al. 2022). To identify candidate enzymes downstream of CL, a BLASTP search was performed using experimentally validated enzymes from various plant species (Table S1) against the predicted proteins of *N. benthamiana* (Ranawaka et al. 2023). Despite the allotetraploid nature of *N. benthamiana*, most enzymes showed only a single clear BLASTP hit (Table S2). For Carlactonoic Acid Methyltransferase (CLAMT), Lateral Branching Oxidoreductase (LBO), and stereoselective BC-ring-forming factor (SRF), a phylogenetic analysis was performed. Based on clustering candidate proteins with enzymes of confirmed enzymatic activity, a single putative SRF and 2 candidates for each of CLAMT and LBO were selected (Figure S1a). To examine the functions of the candidate enzymes, dCasEV2.1 CRISPR-transcriptional activator technology was used, which contains a dCas9–EDLL fusion protein and an MS2–VPR complex recruited via an engineered gRNA scaffold with MS2 aptamers (Selma et al. 2022). A set of gRNAs incorporating this scaffold was designed to target the promoters of specific genes. To assess the efficiency of this technology in activating SL biosynthetic genes, all candidate genes were individually targeted, ie, *More Axillary Growth1* (*NbMAX1*), *NbCYP722C*, *NbSRF1*, *NbCLAMTa*/*b*, *NbLBOa*/*b*, and *NbCYP722A* (Figure S2). Subsequent qRT-PCR analysis revealed transcriptional activation of all of them (Fig. 1a).

**Figure 1:**
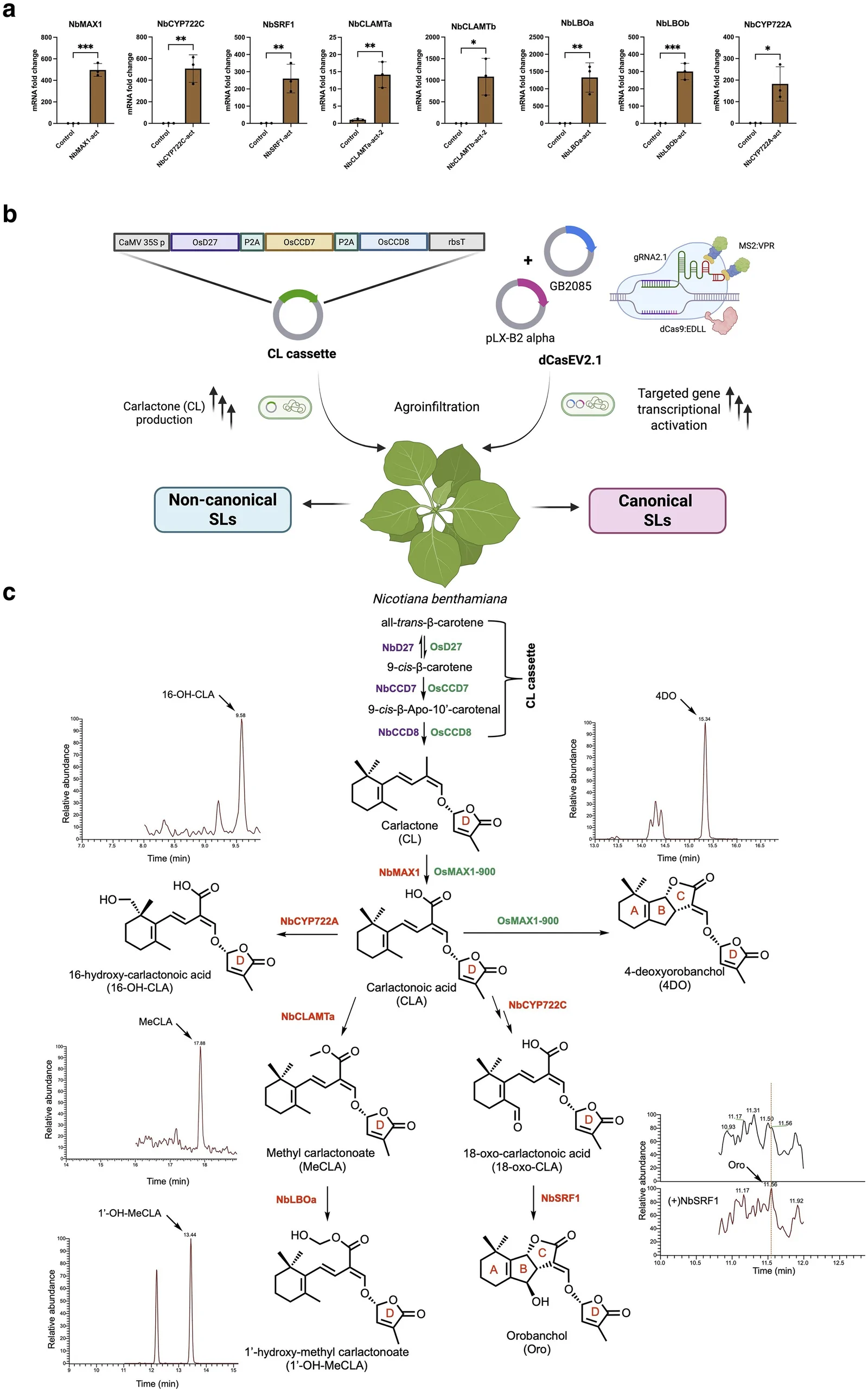
a) Transcriptional activation of SL biosynthesis *N. benthamiana* genes. A gRNA group targeting the *NbDFR* gene was used as a reference. Bars represent average fold change (*n* = 3) ±SD. Three biological replicates, each with 3 technical replicates, were used. Asterisks indicate unpaired Student *t*-test significant values (* = *P* < 0.05, ** = *P* < 0.01, *** = *P* < 0.001). b) System for functionally characterizing *N. benthamiana* genes involved in SL biosynthesis. This figure was generated using BioRender. c) *N. benthamiana* SL pathway. Candidate genes targeted by the dCasEV2.1 activator, with the detection of each metabolite for canonical and noncanonical pathways. *N. benthamiana* genes with reported activity in SL biosynthesis are represented in purple, while genes with a newly described role in the *N. benthamiana* SL biosynthesis are shown in red. Rice genes are represented in green. A representative LC-MS/MS chromatogram for each compound is shown.

To provide the intermediate CL, which is not detectable in leaves, transcriptional activators for *NbD27*, *NbCCD7*, and *NbCCD8* were designed (Figure S1a). These activators were co-expressed with *OsMAX1-900* to produce 4-deoxyorobanchol (4DO) (Fig. 1c) as an indirect measure of CL production efficiency. However, no 4DO was detected (Figure S4a). Since the gene-activation efficiency of the dCasEV2.1 activators decreases with the number of genes simultaneously targeted (Selma et al. 2022), we decided to develop an efficient CL production system independent of the activator technology. A single open reading frame containing the core genes (*OsD27*, *OsCCD7*, and *OsCCD8*) from rice was generated, linked by the F2A peptide (hereafter referred to as the CL-cassette) (Fig. 1b). The combination of the CL-cassette with OsMAX1-900 resulted in high 4DO amounts (Figure S4), confirming the system's efficiency. Hence, the CL-cassette was used to map the *N. benthamiana* SL biosynthetic pathway downstream of CL.

In tomato (*Solanum lycopersicum*), the biosynthesis of orobanchol (Oro) occurs through 18-oxo-carlactonoic acid (18-oxo-CLA), which is produced by CYP722C from CLA. Spontaneous ring closure of 18-oxo-CLA produces 2 enantiomers, *ent*-2′-*epi*-Oro and Oro. However, 18-oxo-CLA is preferentially converted to the Oro enantiomer *in planta* by the activity of the stereospecific factor SRF (Homma et al. 2024). Given that *N. benthamiana* is also a Solanaceae species, we expected similar enzymatic activity from the *N. benthamiana* candidate orthologs. *NbMAX1* and *NbCYP722C* were transcriptionally activated, but no clear *ent-2′-epi-*Oro was obtained and a low amount of Oro was detected under our experimental conditions (Fig. 1c). Nevertheless, the utilization of the transcriptional activator of *NbSRF1* resulted in the production of Oro. (Figure 1c, Figure S5a and b), confirming the function of NbCYP722C and the role of the dirigent protein NbSRF1 in determining stereoselectivity, as in tomato.

The CYP722A enzymes from different plant species, including Arabidopsis, were recently tested in a bacterium-yeast consortium and shown to be involved in noncanonical SL biosynthesis, converting CLA into 16-OH-CLA (Zhou et al. 2025a). To assess if this function is conserved in the *N. benthamiana* ortholog, we transcriptionally activated *NbMAX1* and *NbCYP722A.* Conversely, the formation of 16-OH-CLA was observed (Figs 1c and Figure S6), which indicates the involvement of NbCYP722A in *N. benthamiana's* noncanonical SL biosynthesis.

Regarding the noncanonical pathway, CLAMT-mediated CLA methylation has been reported in both eudicots and monocots (Kuijer et al. 2024; Zhou et al. 2025a). To investigate the involvement of the 2 CLAMT candidates (NbCLAMTa and NbCLAMTb) identified in the BLAST search, 8 activator modules were designed (Table S3). Surprisingly, only high transcriptional enhancement for *NbCLAMTb* was achieved (Fig. 1a, Figure S3c). We assume that the lower activation may be related to the significantly higher basal expression levels of *NbCLAMTa* in leaves compared with other SL genes (Figure S7). The best activator for each gene was implemented (Fig. 1a) and tested for methyl carlactonoate (MeCLA) production. However, none of them showed activity (Figure S8a). To further confirm which NbCLAMT enzyme is involved in SL biosynthesis, the corresponding coding sequences (CDS) of each NbCLAMT, instead of the CRISPR activators, were transiently expressed in combination with CL-cassette and *NbMAX1* activator. Surprisingly, NbCLAMTa can convert CLA to MeCLA efficiently (Figure S8a and b). However, NbCLAMTb showed low methylation activity toward CLA under our experimental conditions (Figure S8a and b). Although NbCLAMTb shares high protein identity with NbCLAMTa, it appears not to play a major role in *N. benthamiana* SL biosynthesis. Alternatively, NbCLAMTb might methylate an unknown SL different from CLA.

The enzyme LBO, a 2-oxoglutarate-dependent dioxygenase, oxidizes MeCLA to 1′-OH-MeCLA in the noncanonical SL biosynthetic pathway. However, in vitro assays showed that LBO from Arabidopsis, catalyzes the demethylation of MeCLA into CLA at a higher rate than the hydroxylation of MeCLA into 1′-OH-MeCLA (Yoneyama et al. 2020). Therefore, the precise function of LBO *in planta* remains unclear, although expression of AtLBO in a MeCLA-producing microbial consortium suggests that AtLBO functions as an oxidase, rather than a demethylase (Zhou et al. 2025a). To characterize the NbLBO function, activator modules for *NbLBOa* and *NbLBOb* were generated and co-expressed with the MeCLA module (CL-cassette, *NbMAX1*-activator, and *NbCLAMTa-*CDS). 1′-OH-MeCLA was detected upon the activation of *NbLBOa* transcription (Figure S9a), although not all MeCLA was consumed (Figure S9b). To determine whether the incomplete conversion was due to low expression, *NbLBOa*-CDS was transiently expressed rather than transcriptionally activated. Subsequent Liquid Chromatography-Mass Spectrometry (LC-MS) analysis revealed complete hydroxylation of MeCLA to 1′-OH-MeCLA, indicating that LBO functions as an oxygenase rather than a demethylase *in planta* (Figure S9a and b). The second LBO candidate enzyme, NbLBOb, did not show any activity upon either transcriptional activation or transient expression (Figure S9a and b). A possible explanation is that it results from a truncation of the double-stranded β-helix (Figure S1b).

In conclusion, we characterized several enzymes involved in SL biosynthesis in *N. benthamiana* by developing an expression cassette (Fig. 1b) that enables high-yield production of CL. This approach makes the characterization of downstream enzymes more efficient by providing a high amount of precursor for downstream products and reducing the number of plasmids used for co-infiltration. Our results identify the contribution of the *NbCLAMT* gene in SL biosynthesis and clarify the enzymatic function of NbLBO *in planta*. *N. benthamiana* leaves provide a low-background system in which SL-related products detected following transient expression can be attributed to the introduced enzymes rather than to the endogenous enzymes. This property underlies the widespread use of the system to assign functions to SL candidate pathway enzymes, with these assignments subsequently supported by SL profiling and loss-of-function phenotypes in the source species (Zhang et al. 2018; Yoda et al. 2021; Kuijer et al. 2024; Li et al. 2024). We therefore expect the enzymatic activities established here to reflect the true biosynthetic capacity of the corresponding *N. benthamiana* enzymes. Additionally, we demonstrate that the dCasEV2.1 activator technology can be utilized to study complex pathways, such as the SL pathway. This is the first time that this tool has been used to investigate SL biosynthesis. Like any method, the technology has its limitations, as demonstrated by the fact that not all genes can be activated and that the stack of activators can reduce their efficiency. Nevertheless, the dCasEV2.1 activator is a valuable tool for gene discovery and characterization in *N. benthamiana*. It could also be used to quickly screen other pathways and identify new candidates from transcriptomic experiments performed directly, due to its simple design and efficiency.

## Supplementary Material

kiag748_Supplementary_Data

## Data Availability

The data underlying this article are available in the article and in its online supplementary material.

## References

[kiag748-B1] Homma M et al 2024. Insights into stereoselective ring formation in canonical strigolactone: identification of a dirigent domain-containing enzyme catalyzing orobanchol synthesis. Proc Natl Acad Sci USA. 121:e2313683121. 10.1073/pnas.2313683121. PMC11214005 38905237

[kiag748-B2] Jia KP, Baz L, Al-Babili S. 2018. From carotenoids to strigolactones. J Exp Bot. 69:2189–2204. 10.1093/jxb/erx476. 29253188

[kiag748-B3] Kuijer HNJ et al 2024. Chromosome-scale pearl millet genomes reveal CLAMT1b as key determinant of strigolactone pattern and striga susceptibility. Nat Commun. 15:6906. 10.1038/s41467-024-51189-w. PMC11319436 39134551

[kiag748-B4] Li C et al 2024. OsCYP706C2 diverts rice strigolactone biosynthesis to a noncanonical pathway branch. Sci Adv. 10:eadq3942. 10.1126/sciadv.adq3942. PMC11352842 39196928

[kiag748-B5] Ranawaka B et al 2023. A multi-omic Nicotiana benthamiana resource for fundamental research and biotechnology. Nat Plants. 9:1558–1571. 10.1038/s41477-023-01489-8. PMC10505560 37563457

[kiag748-B6] Selma S et al 2022. Custom-made design of metabolite composition in N. benthamiana leaves using CRISPR activators. Plant Biotechnol J. 20:1578–1590. 10.1111/pbi.13834. PMC9342607 35514036

[kiag748-B7] Wang JY, Chen GTE, Braguy J, Al-Babili S. 2024. Distinguishing the functions of canonical strigolactones as rhizospheric signals. Trends Plant Sci. 29:925–936. 10.1016/j.tplants.2024.02.013. 38521698

[kiag748-B8] Yata A, Nosaki S, Yoda A, Nomura T, Miura K. 2022. Production and stably maintenance of strigolactone by transient expression of biosynthetic enzymes in Nicotiana benthamiana. Front Plant Sci. 13:1027004. 10.3389/fpls.2022.1027004. PMC9650523 36388605

[kiag748-B9] Yoda A et al 2021. Strigolactone biosynthesis catalyzed by cytochrome P450 and sulfotransferase in sorghum. New Phytol. 232:1999–2010. 10.1111/nph.17737. PMC9292024 34525227

[kiag748-B10] Yoneyama K et al 2020. Hydroxyl carlactone derivatives are predominant strigolactones in Arabidopsis. Plant Direct. 4:e00219. 10.1002/pld3.219. PMC7207163 32399509

[kiag748-B11] Zhang Y et al 2018. The tomato MAX1 homolog, SlMAX1, is involved in the biosynthesis of tomato strigolactones from carlactone. New Phytol. 219:297–309. 10.1111/nph.15131. 29655242

[kiag748-B12] Zhou A et al 2025a. Evolution of interorganismal strigolactone biosynthesis in seed plants. Science. 387:eadp0779. 10.1126/science.adp0779. 39818909

[kiag748-B13] Zhou Q et al 2025b. Chemistry and chemical biology tools contributing to the discovery and functional characterization of strigolactones. Front Plant Sci. 16:1618437. 10.3389/fpls.2025.1618437. PMC12213501 40606478

