# Supplementary material for: CRISPR-based transcriptional activation deciphers strigolactone biosynthesis in Nicotiana benthamiana
Source: Plant Physiol. 2026 Oct 6;202(2):kiag748. doi: 10.1093/plphys/kiag748 (PMC13677784; doi:10.1093/plphys/kiag748)
Supplement: Supplementary file 1 [file kiag748_supplementary_data.zip › PlantPhys_Supplementary_Figures_Reviewers_R2.pdf]

Supplementary Figures

Figure S1. *N. benthamiana* CLAMT, LBO, and SRF candidate orthologs. (a) Phylogenetic trees for the CLAMT, LBO, and SRF candidates in *N. benthamiana*. Candidate proteins in *N. benthamiana* are represented in green, and the reference proteins from other species in orange. Phylogenetic trees were constructed using RaXML with 1000 bootstrap replicates. (b) Protein structure of AtLBO and NbLBOs. The purple region in the scheme represents the double-stranded ß-helix (DSBH). The DSBH sequence of the proteins is shown, and the essential amino acids for LBO catalytic activity are highlighted in AtLBO.

Figure S2. Different gRNAs targeting the promoter of *N. benthamiana* candidate genes. The distance is estimated using the transcription starting site (TSS) as a reference.

Figure S3. Transcriptional activation of different *N. benthamiana* candidate genes. Bars represent average fold change (n=3) +/- SD. Three biological replicates, each with three technical replicates, were used. Asterisks indicate unpaired Student t-test significant values (* = $P < 0.05$, ** = $P < 0.01$, *** = $P < 0.001$). (a) Activation of the core genes responsible for CL production. (b) Efficiency of different *NbCLAMTa* activators. (c) Efficiency of different *NbCLAMTb* activators.

Figure S4. CL production evaluation using 4DO production (a) Multiple Reaction Monitoring (MRM) analysis of 4DO production in *N. benthamiana* leaves transiently expressing *OsMAX1-900* in combination with the NbD27act, NbCCD7act and NbCCD8act, or with the CL-cassette. Three biological replicates were used. (b) 4DO quantification represented as picograms (pg) of the compound per milligram (mg) of leaf dry weight (DW).

Figure S5. Oro biosynthesis analysis in *N. benthamiana.* (a) MRM of the activity of NbCYP722C and NbSRF1. The Oro rice pathway using *OsMAX1-900* and *OsMAX1-1400* was used as a control. Three biological replicates were used. A representative LC-MS/MS chromatogram for each compound is shown (b) Oro quantification represented as pg of the compound per mg of leaf (DW).

Figure S6. MRM of the activity of NbCYP722A. Three biological replicates were used. A representative LC-MS/MS chromatogram for each compound is shown.

Figure S7. Basal expression levels of *N. benthamiana* candidate genes. Bars represent average fold change (n=3) +/- SD. Three biological replicates, each with three technical replicates, were used. Asterisks indicate a one-way ANOVA test (**** = $P < 0.0001$). Only the significant differences of *NbCLAMTa* relative to the other genes are displayed.

Figure S8. NbCLAMTs enzymatic activity. (a) MRM of the activity of NbCLAMTa and NbCLAMTb. Three biological replicates were used. A representative LC-MS/MS chromatogram for each compound is shown. (b) MeCLA quantification using the combinations that involved CLAMTs transiently expressed with a pBIN plasmid. Quantification is represented as pg of the compound per mg of leaf DW.

Figure S9. MRM of the activity of NbLBOa and NbLBOb. Three biological replicates were used. A representative LC-MS/MS chromatogram for each compound is shown.

a

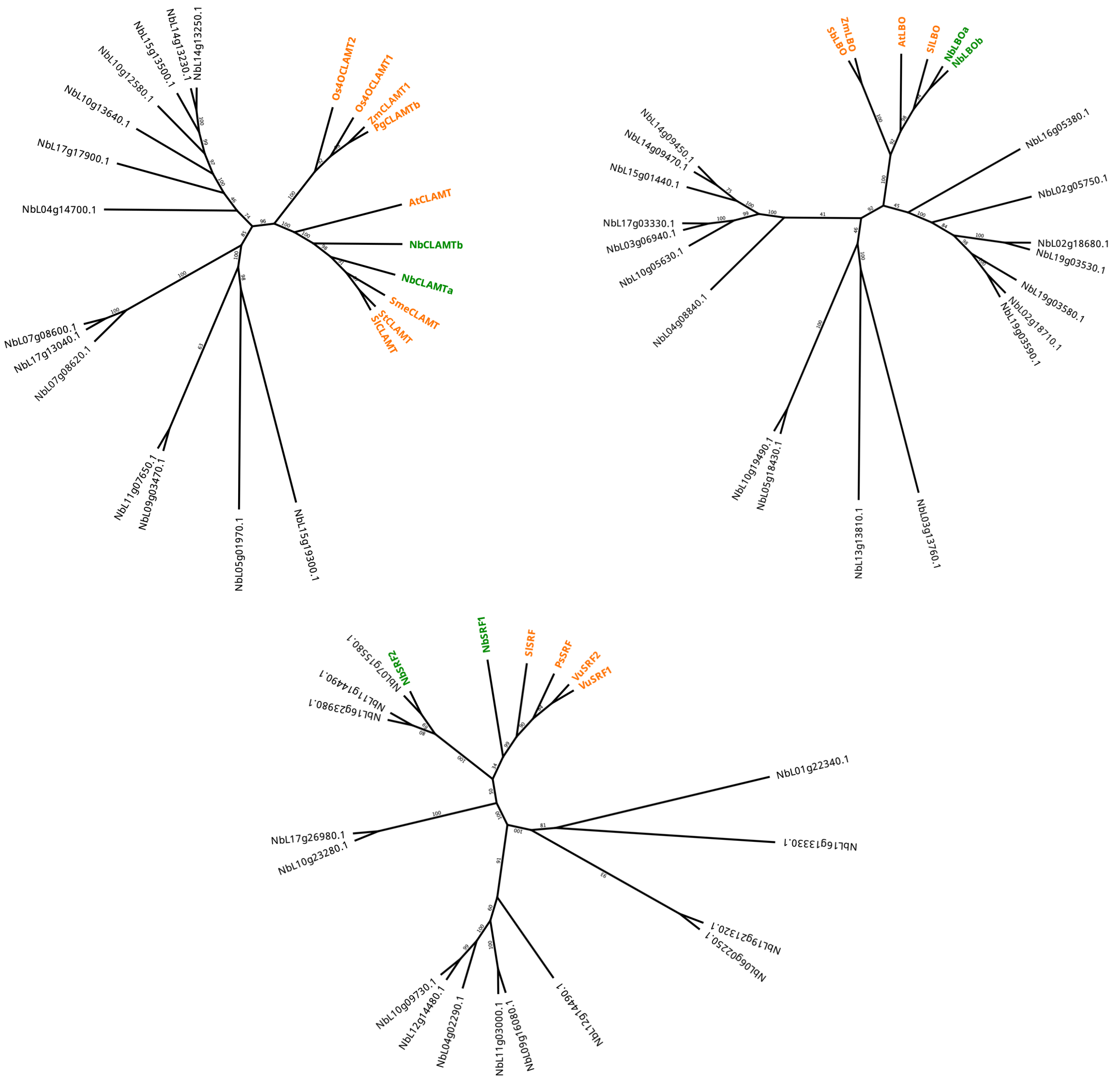

b

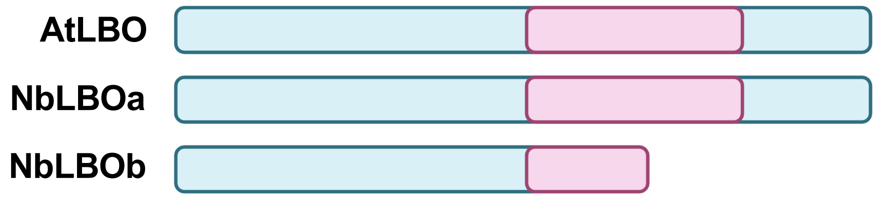

AtLBO F E E M F G E A V Q A V R M N Y Y P P C S S P D L V L G L S P H S D G S A L T V L Q Q S K N S C V G L Q I L K D N T W V P V K
NbLBOa F E E M F G V A V Q A L R M N Y Y P A C P R P D L V L G L S P H S D G S A L T V L Q Q G K G N S V G L Q I L K D N V W V P I Q
NbLBOb F E D M F G V A V Q A L R M N Y Y P A C P R L D L V L G L S P H L D G S A L T V L Q Q G K G S S V G L Q I L K D N V W V P I *

AtLBO P L P N A L V I N I G D T I E V L S N G K Y K S V E H R A V T N R E K E R L T I V T F Y A P N Y
NbLBOa P V P N A L V I N I G D T I E V L T N G R Y K S V E H R A V T H Q E K D R L S I V T F Y A P S Y
NbLBOb

Figure S1

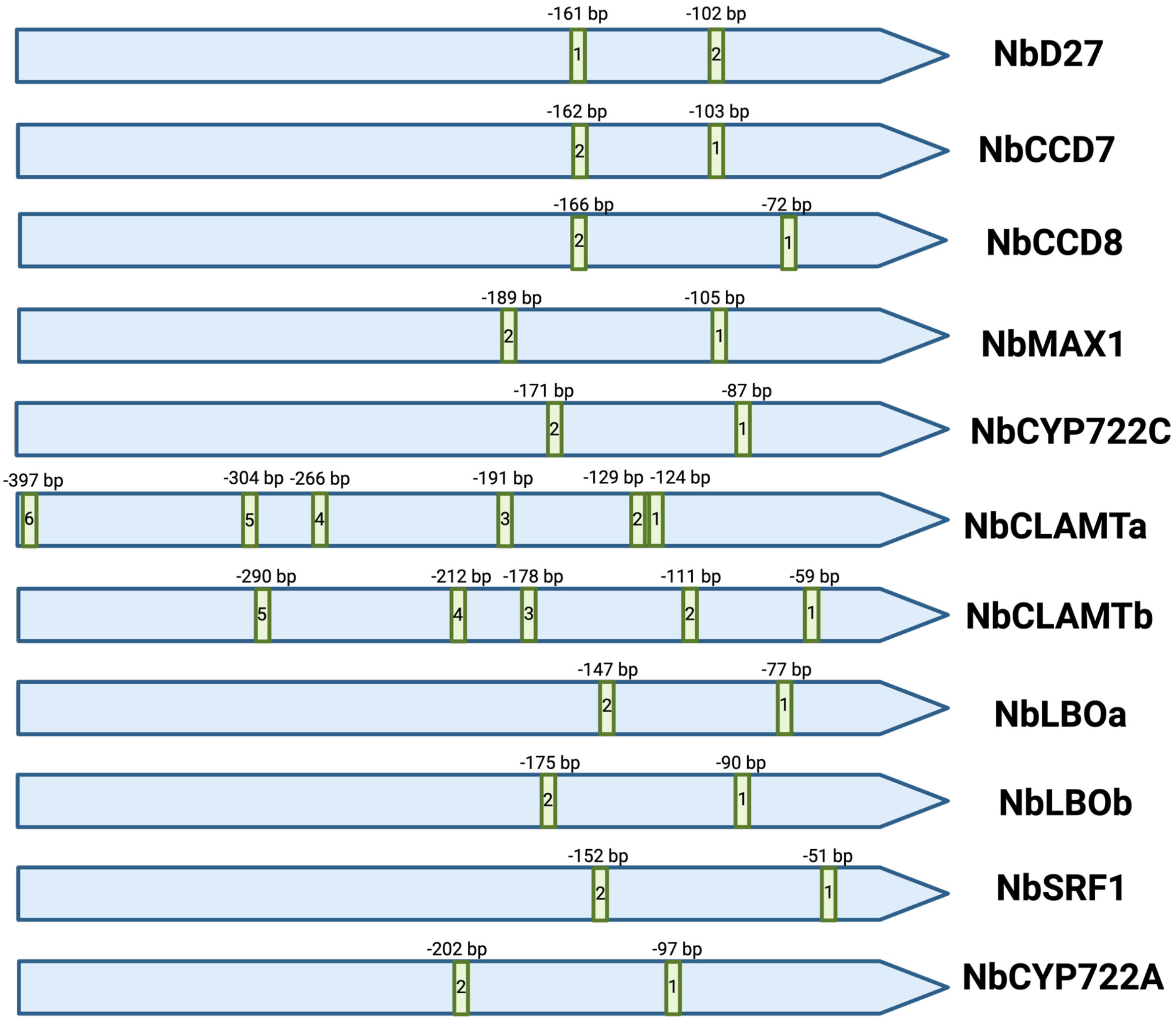
-161 bp
-102 bp
1
2
NbD27
-162 bp
-103 bp
2
1
NbCCD7
-166 bp
-72 bp
2
1
NbCCD8
-189 bp
-105 bp
2
1
NbMAX1
-171 bp
-87 bp
2
1
NbCYP722C
-397 bp
-304 bp
-266 bp
-191 bp
-129 bp
-124 bp
6
5
4
3
2
1
NbCLAMTa
-290 bp
-212 bp
-178 bp
-111 bp
-59 bp
5
4
3
2
1
NbCLAMTb
-147 bp
-77 bp
2
1
NbLBOa
-175 bp
-90 bp
2
1
NbLBOb
-152 bp
-51 bp
2
1
NbSRF1
-202 bp
-97 bp
2
1
NbCYP722A

Figure S2

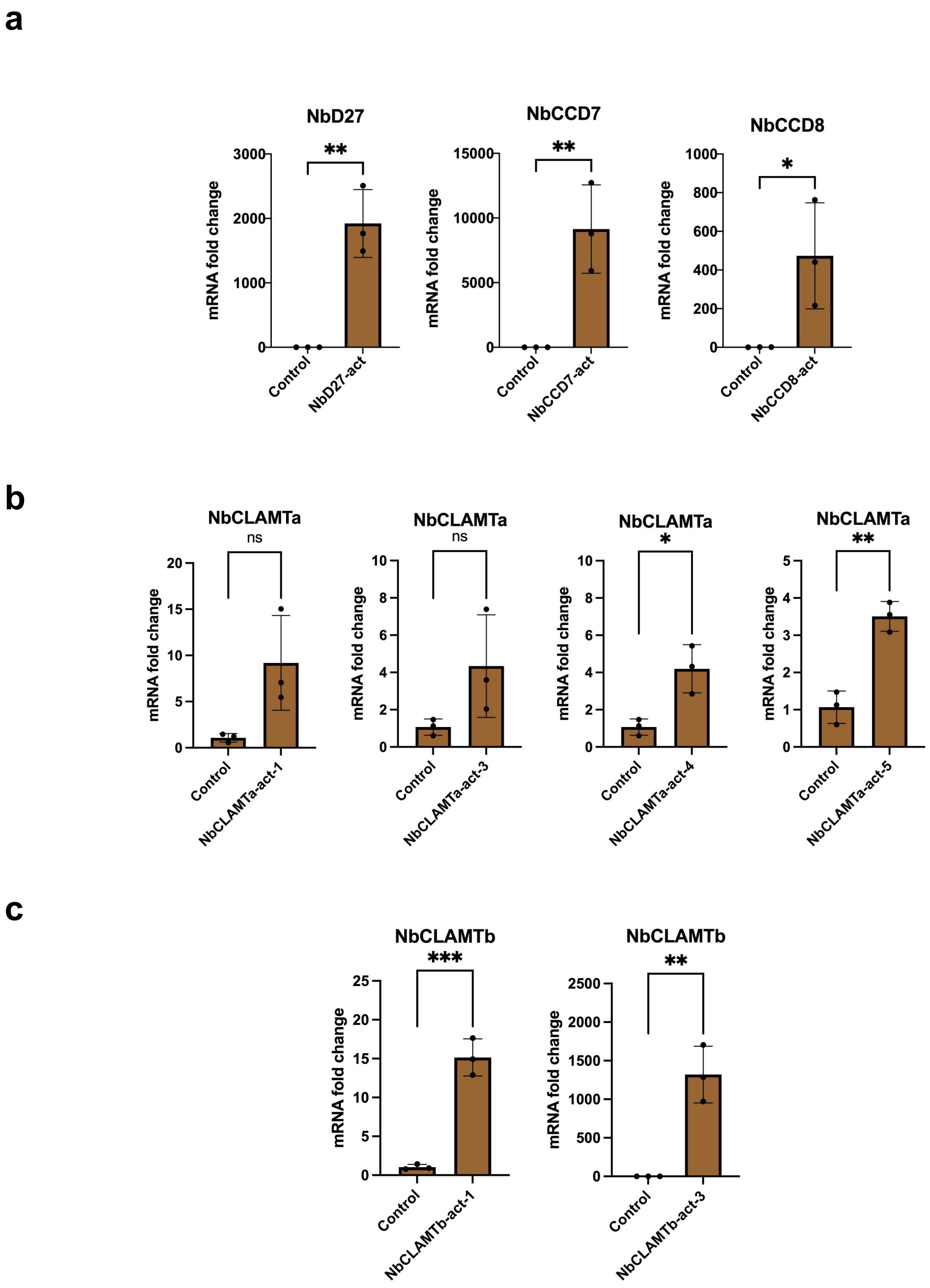
a
NbD27
**
mRNA fold change
3000
2000
1000
0
Control
NbD27-act
NbCCD7
**
15000
10000
5000
NbCCD7-act
NbCCD8
*
1000
800
600
400
200
NbCCD8-act
b
NbCLAMTa
ns
20
15
10
5
NbCLAMTa-act-1
NbCLAMTa-act-3
NbCLAMTa-act-4
NbCLAMTa-act-5
c
NbCLAMTb
***
25
2500
2000
1500
1000
500
NbCLAMTb-act-1
NbCLAMTb-act-3

Figure S3

a

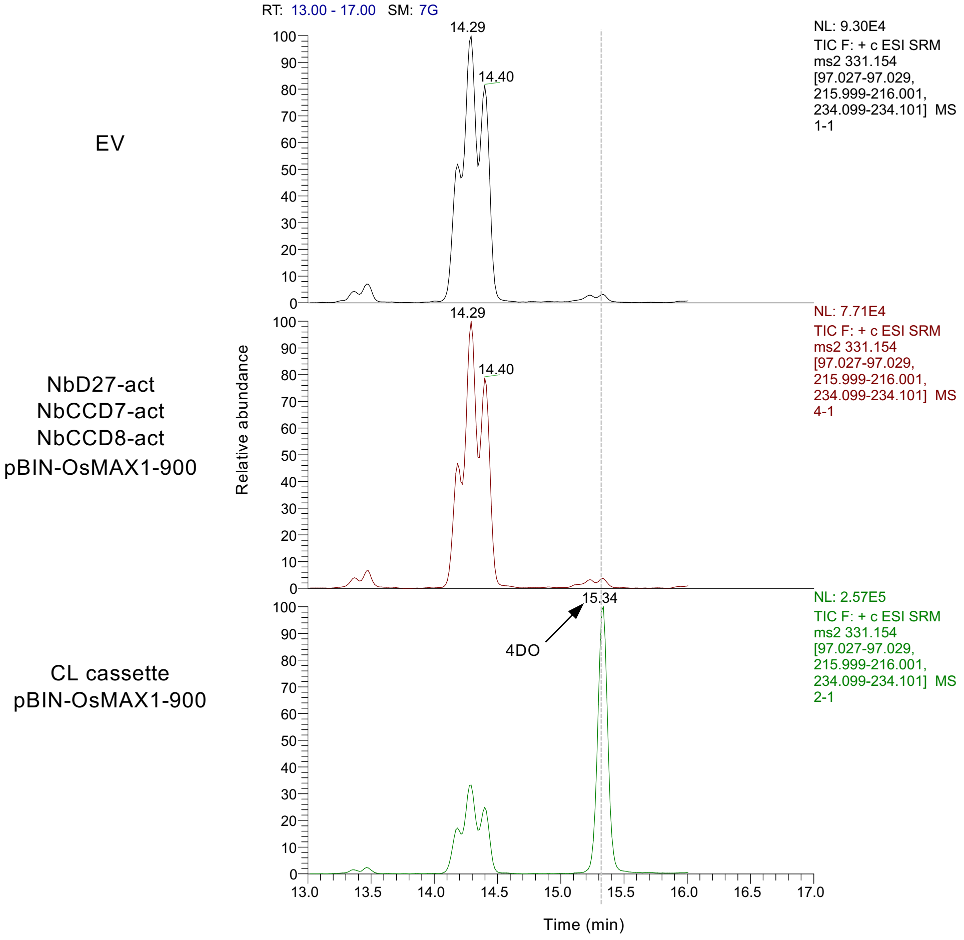

RT: 13.00 - 17.00 SM: 7G
EV
NbD27-act
NbCCD7-act
NbCCD8-act
pBIN-OsMAX1-900
CL cassette
pBIN-OsMAX1-900
14.29
14.40
15.34
4DO
NL: 9.30E4
TIC F: + c ESI SRM ms2 331.154 [97.027-97.029, 215.999-216.001, 234.099-234.101] MS 1-1
NL: 7.71E4
TIC F: + c ESI SRM ms2 331.154 [97.027-97.029, 215.999-216.001, 234.099-234.101] MS 4-1
NL: 2.57E5
TIC F: + c ESI SRM ms2 331.154 [97.027-97.029, 215.999-216.001, 234.099-234.101] MS 2-1
Relative abundance
Time (min)

b

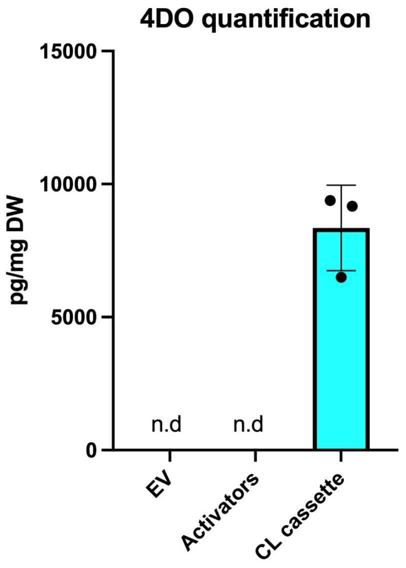

4DO quantification
pg/mg DW
15000
10000
5000
0
n.d
n.d
EV
Activators
CL cassette

Figure S4

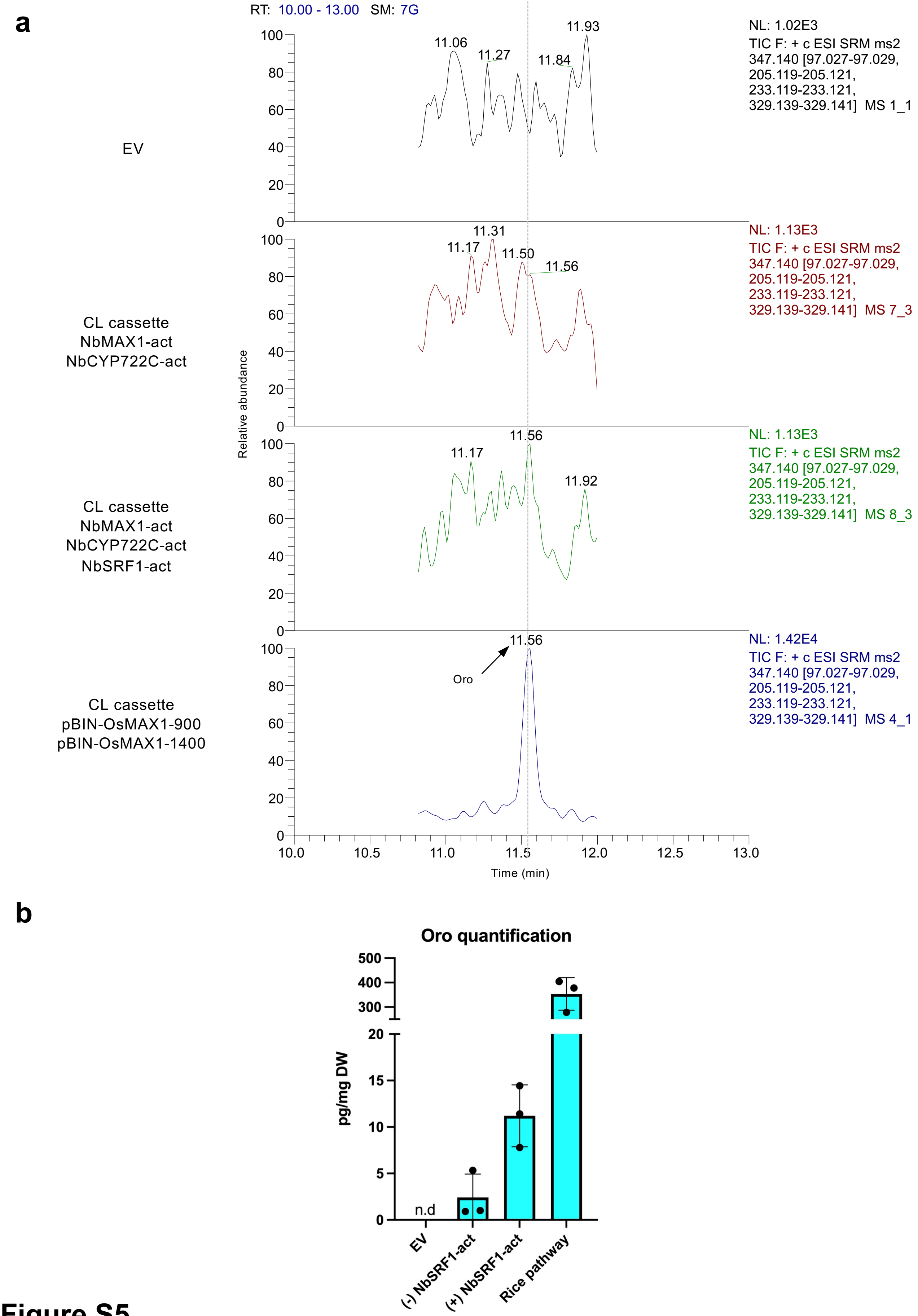
a
RT: 10.00 - 13.00 SM: 7G
EV
CL cassette
NbMAX1-act
NbCYP722C-act
CL cassette
NbMAX1-act
NbCYP722C-act
NbSRF1-act
CL cassette
pBIN-OsMAX1-900
pBIN-OsMAX1-1400
NL: 1.02E3
TIC F: + c ESI SRM ms2 347.140 [97.027-97.029, 205.119-205.121, 233.119-233.121, 329.139-329.141] MS 1_1
NL: 1.13E3
TIC F: + c ESI SRM ms2 347.140 [97.027-97.029, 205.119-205.121, 233.119-233.121, 329.139-329.141] MS 7_3
NL: 1.13E3
TIC F: + c ESI SRM ms2 347.140 [97.027-97.029, 205.119-205.121, 233.119-233.121, 329.139-329.141] MS 8_3
NL: 1.42E4
TIC F: + c ESI SRM ms2 347.140 [97.027-97.029, 205.119-205.121, 233.119-233.121, 329.139-329.141] MS 4_1
Oro
Relative abundance
Time (min)
b
Oro quantification
pg/mg DW
n.d
EV
(-) NbSRF1-act
(+) NbSRF1-act
Rice pathway

Figure S5

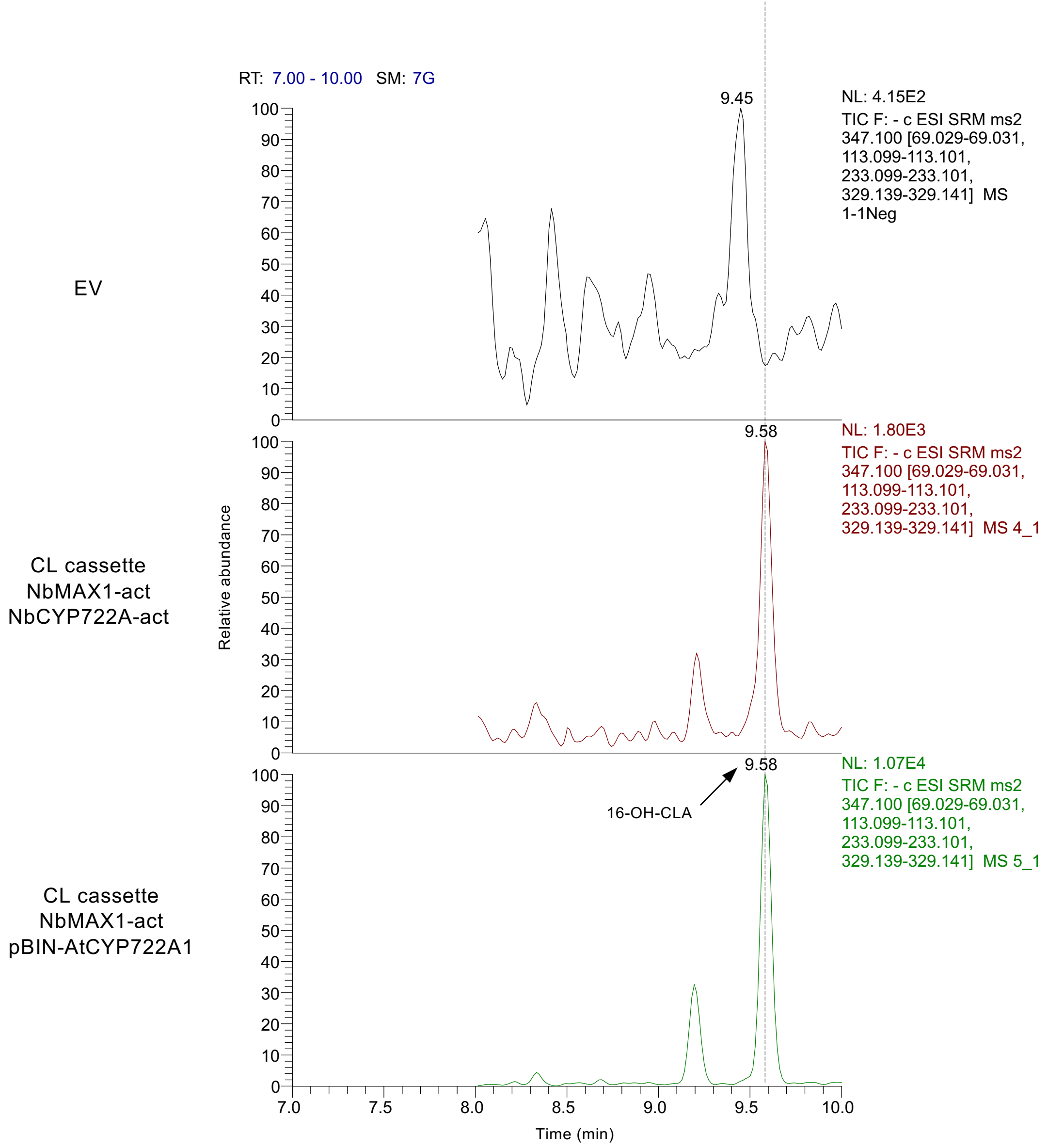
RT: 7.00 - 10.00 SM: 7G
EV
CL cassette NbMAX1-act NbCYP722A-act
CL cassette NbMAX1-act pBIN-AtCYP722A1
Relative abundance
Time (min)
9.45
9.58
9.58
16-OH-CLA
NL: 4.15E2
TIC F: - c ESI SRM ms2 347.100 [69.029-69.031, 113.099-113.101, 233.099-233.101, 329.139-329.141] MS 1-1Neg
NL: 1.80E3
TIC F: - c ESI SRM ms2 347.100 [69.029-69.031, 113.099-113.101, 233.099-233.101, 329.139-329.141] MS 4_1
NL: 1.07E4
TIC F: - c ESI SRM ms2 347.100 [69.029-69.031, 113.099-113.101, 233.099-233.101, 329.139-329.141] MS 5_1

Figure S6

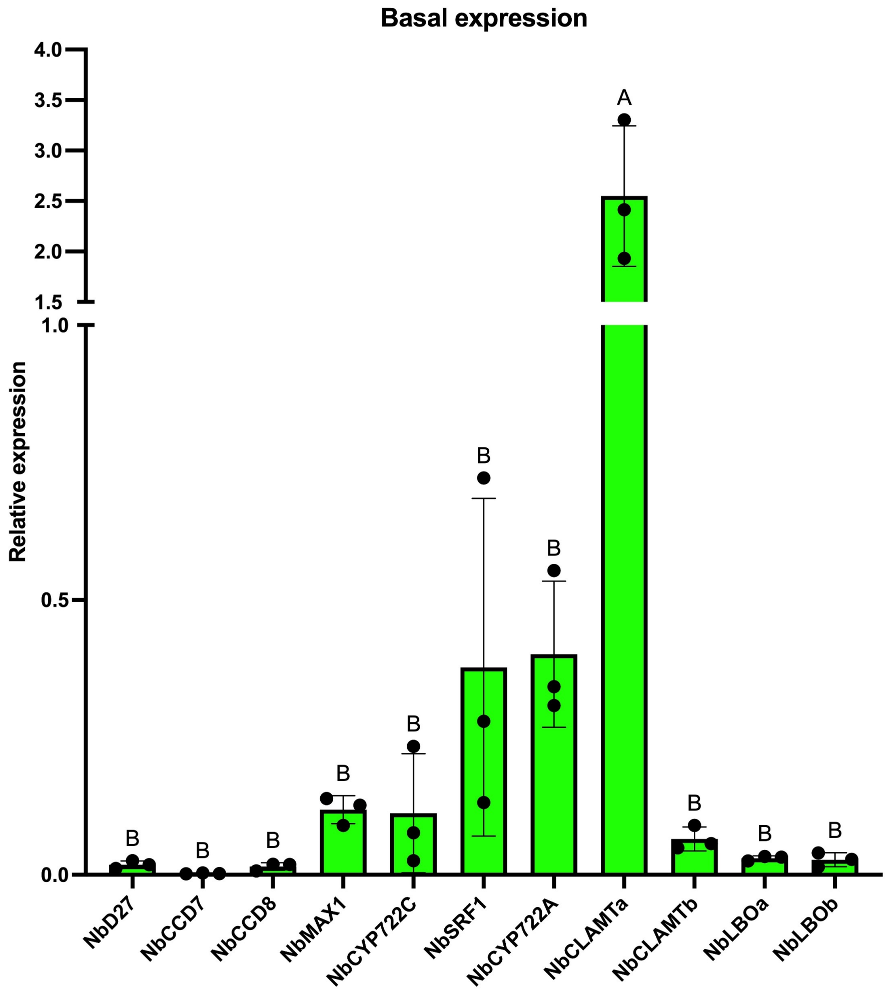
Basal expression
Relative expression
4.0
3.5
3.0
2.5
2.0
1.5
1.0
0.5
0.0
A
B
NbD27
NbCCD7
NbCCD8
NbMAX1
NbCYP722C
NbSRF1
NbCYP722A
NbCLAMTa
NbCLAMTb
NbLBOa
NbLBOb

Figure S7

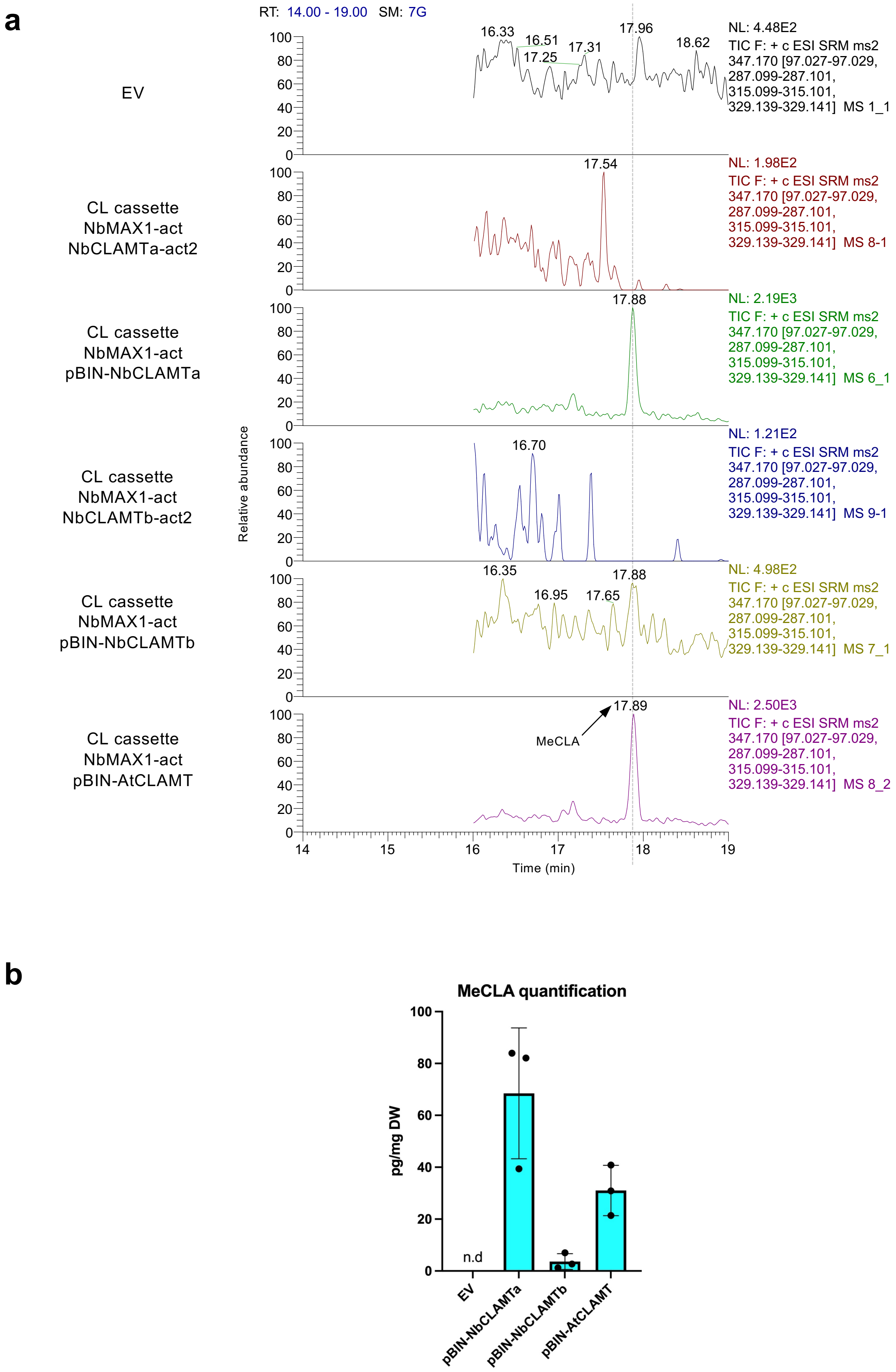

a
RT: 14.00 - 19.00 SM: 7G
EV
NL: 4.48E2
TIC F: + c ESI SRM ms2 347.170 [97.027-97.029, 287.099-287.101, 315.099-315.101, 329.139-329.141] MS 1_1
CL cassette NbMAX1-act NbCLAMTa-act2
NL: 1.98E2
TIC F: + c ESI SRM ms2 347.170 [97.027-97.029, 287.099-287.101, 315.099-315.101, 329.139-329.141] MS 8-1
CL cassette NbMAX1-act pBIN-NbCLAMTa
NL: 2.19E3
TIC F: + c ESI SRM ms2 347.170 [97.027-97.029, 287.099-287.101, 315.099-315.101, 329.139-329.141] MS 6_1
CL cassette NbMAX1-act NbCLAMTb-act2
NL: 1.21E2
TIC F: + c ESI SRM ms2 347.170 [97.027-97.029, 287.099-287.101, 315.099-315.101, 329.139-329.141] MS 9-1
CL cassette NbMAX1-act pBIN-NbCLAMTb
NL: 4.98E2
TIC F: + c ESI SRM ms2 347.170 [97.027-97.029, 287.099-287.101, 315.099-315.101, 329.139-329.141] MS 7_1
CL cassette NbMAX1-act pBIN-AtCLAMT
NL: 2.50E3
TIC F: + c ESI SRM ms2 347.170 [97.027-97.029, 287.099-287.101, 315.099-315.101, 329.139-329.141] MS 8_2
MeCLA
Relative abundance
Time (min)
b
MeCLA quantification
pg/mg DW
n.d
EV
pBIN-NbCLAMTa
pBIN-NbCLAMTb
pBIN-AtCLAMT

Figure S8

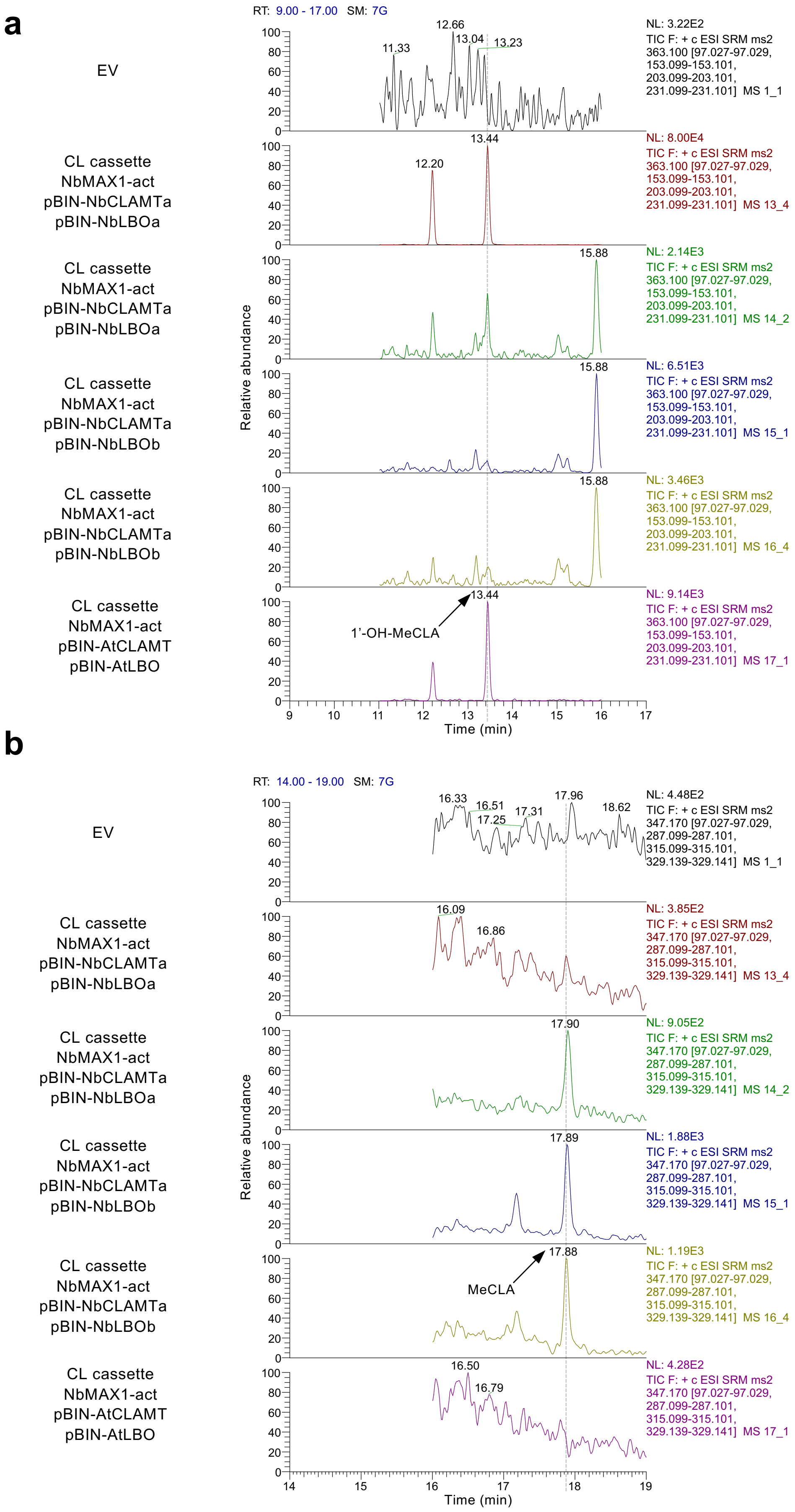
a
b
EV
CL cassette
NbMAX1-act
pBIN-NbCLAMTa
pBIN-NbLBOa
pBIN-NbLBOb
pBIN-AtCLAMT
pBIN-AtLBO
RT: 9.00 - 17.00 SM: 7G
RT: 14.00 - 19.00 SM: 7G
1'-OH-MeCLA
MeCLA
Relative abundance
Time (min)
NL: 3.22E2
NL: 8.00E4
NL: 2.14E3
NL: 6.51E3
NL: 3.46E3
NL: 9.14E3
TIC F: + c ESI SRM ms2 363.100 [97.027-97.029, 153.099-153.101, 203.099-203.101, 231.099-231.101]
NL: 4.48E2
NL: 3.85E2
NL: 9.05E2
NL: 1.88E3
NL: 1.19E3
NL: 4.28E2
TIC F: + c ESI SRM ms2 347.170 [97.027-97.029, 287.099-287.101, 315.099-315.101, 329.139-329.141]
MS 1_1
MS 13_4
MS 14_2
MS 15_1
MS 16_4
MS 17_1

Figure S9
